# Activity of Amphotericin B and Anidulafungin Combined with Rifampicin, Clarithromycin, Ethylenediaminetetraacetic Acid, *N*-Acetylcysteine, and Farnesol against *Candida tropicalis* Biofilms

**DOI:** 10.3390/jof3010016

**Published:** 2017-03-22

**Authors:** Marcelo Ernesto Fernández-Rivero, José L. del Pozo, Amparo Valentín, Araceli Molina de Diego, Javier Pemán, Emilia Cantón

**Affiliations:** 1 Departamento de Microbiología, Universidad de Navarra, 31008 Pamplona, Spain; marcelo_fernandez@iislafe.es (M.E.F.-R.); jdelpozo@unav.es (J.L.d.P.); 2 Laboratorio de Biofilms Microbianos, Clínica Universidad de Navarra, 31008 Pamplona, Spain; 3 Grupo de Infección Grave, Instituto de Investigación Sanitaria La Fe, 46026 Valencia, Spain; valentin_amp@gva.es (A.V.); molina_ara@gva.es (A.M.d.D.); canton_emi@gva.es (E.C.); 4 Área de Enfermedades Infecciosas, Clínica Universidad de Navarra, 31008 Pamplona, Spain; 5 Servicio de Microbiología Clínica, Hospital Universitario La Fe, 46026 Valencia, Spain

**Keywords:** *Candida tropicalis*, CDC Biofilm Reactor, biofilm, amphotericin B, anidulafungin, PTFE, titanium, anti-biofilm compounds

## Abstract

We evaluated the activity of (1) amphotericin-B (AMB), combined with rifampicin (RIF), clarithromycin (CLA), *N*-acetylcysteine (NAC), ethylenediaminetetraacetic acid (EDTA), and farnesol (FAR) (1000, 1000, 1000, 4000, and 30,000 mg/L, and 300 µM, respectively), against *Candida tropicalis* biofilms formed on polytetrafluoroethylene (PTFE) and (2) anidulafungin (ANF) combined with the same compounds at 8, 10, 5, 40, and 30 mg/L, and 30 µM, respectively, against biofilms formed on titanium. Biofilm growth kinetics were performed in a CDC Biofilm Reactor (CBR). PTFE or titanium disks were removed from the CBR at 24, 48, 72, and 96 h to determine the Log_10_CFU/cm^2^. Killing kinetics were performed by adding the drugs to 24-h-mature biofilms (time 0). Disks were removed after 24, 48, and 72 h of drug exposure to determine Log_10_CFU/cm^2^. Viable cells in biofilms were 4.73 and 4.29 Log_10_CFU/cm^2^ on PTFE and titanium, respectively. Maximum Log_10_ decreases in CFU/cm^2^ depend on the combination and were: 3.53 (AMB + EDTA), 2.65 (AMB + RIF), 3.07 (AMB + NAC), 2.52 (AMB + CLA), 1.49 (AMB + FAR), 2.26 (ANF + EDTA), 2.45 (ANF + RIF), 2.47 (ANF + NAC), 1.52 (ANF + CLA), and 0.44 (ANF + FAR). In conclusion, EDTA, NAC, RIF, and CLA improve the activity of AMB and ANF against biofilms developed on both surfaces, which could be an effective strategy against *C. tropicalis* biofilm-related infections.

## 1. Introduction

Biomedical devices are essential for treatment of a number of diseases, substantially improving the quality of life and survival of patients. Several materials are commonly used in the manufacture of these devices. Plastic polymers such as polytetrafluoroethylene (PTFE, commercially known as Teflon^®^) are hydrophobic materials commonly used for the manufacture of catheters. On the other hand, about 80% of hip and knee prostheses are manufactured using metallic biomaterials such as titanium (a hydrophilic surface), which, due to its excellent biocompatibility, has gained importance in the last few years. However, *Candida* spp. are able to attach to biomaterials and tissues, and once an organism colonizes the surface of a device it forms a complex microbial community named a biofilm that grows embedded in a polymeric extracellular matrix that is regulated by quorum-sensing molecules [1]. These biofilms are exposed to a wide range of drug concentrations during treatment. For example, biofilms formed on catheters are exposed to high concentrations during the antimicrobial-lock therapy (ALT) [2], while biofilms formed on a prosthesis are exposed to drug concentrations usually reached in serum after the administration of standard doses [3]. *Candida* spp. biofilm cells have proved to be more resistant to conventional antifungal agents than planktonic cells, and several authors have observed that this resistance especially affects azoles [4]. Furthermore, some species such as *Candida tropicalis* also have reduced susceptibility to echinocandins when grown in a biofilm [5].

Various strategies have been proposed for the prevention and treatment of *Candida* infections related to biomedical devices—for example, the use of antimicrobial agents combined with anti-biofilm compounds such as chelating agents, matrix disrupters, quorum sensing molecules, and antibiotics. Among these substances, ethylenediaminetetraacetic acid (EDTA), a calcium-chelating agent, inhibits the transport and assemblage of cell wall components and also affects the morphogenesis of *Candida albicans* by altering the calcium-dependent calmodulin modulations [6]. *N*-acetylcysteine (NAC), is a mucolytic and antioxidant agent that reduces cellular adherence and extracellular matrix production in bacterial biofilms [7]. Rifampicin (RIF) and clarithromycin (CLA) are two antibiotics that inhibit the bacterial RNA polymerase and ribosome, respectively. It has been reported that RIF also displays activity against the fungal RNA polymerase, and CLA induces the activity of enzymes such as hexosaminidases, which could affect hexosamines, an important component of the *C. tropicalis* biofilm matrix [8]. Farnesol (FAR) is a quorum-sensing molecule that is self-produced by *C. albicans* biofilms as a response to high cell densities. It inhibits the yeast–hyphae transition and disrupts biofilms to prevent nutrient depletion by overpopulation [9]. The use of these compounds in combination with antifungal agents could be an effective strategy to prevent and/or eradicate the *C. tropicalis* biofilm formed on the surface of biomedical devices. 

The aim of our study was to evaluate the activity of amphotericin B (AMB) and anidulafungin (ANF) alone and combined with RIF, CLA, NAC, EDTA, and FAR, against *C. tropicalis* biofilms formed on PTFE and titanium, using the CDC Biofilm Reactor (CBR) as an in vitro model.

## 2. Materials and Methods

### 2.1. Drugs and Concentrations Assayed

AMB, EDTA, NAC, and FAR were provided by Sigma-Aldrich (Madrid, Spain) and dissolved in dimethyl sulfoxide (AMB, FAR) and water (EDTA, NAC). ANF (Pfizer, Madrid, Spain), RIF (Sanofi-Aventis, Barcelona, Spain) and CLA (Abbott, Madrid, Spain) were provided as pure powders by the manufacturers and dissolved in dimethyl sulfoxide (ANF) and water (RIF, CLA). Stock solutions were stored at −80 °C until use within one year. The maximum DMSO concentration in the CBR was always ≤1%.

Concentrations assayed against biofilms formed on PTFE were those potentially used in ALT: AMB 1000 mg/L, RIF 1000 mg/L, CLA 1000 mg/L, EDTA 30,000 mg/L, NAC 4000 mg/L, and FAR 300 µM [10,11,12,13]. Against biofilms formed on titanium, the concentrations assayed were chosen taking into account the peak serum concentration reached after the administration of standard doses: ANF 8 mg/L, RIF 10 mg/L, CLA 5, EDTA 30 mg/L, NAC 40 mg/L, and FAR 30 µM [14,15,16]. Concentrations of EDTA and FAR were chosen since they have been used by other authors [9,17].

### 2.2. CBR and Biomaterials

**Figure 1:**
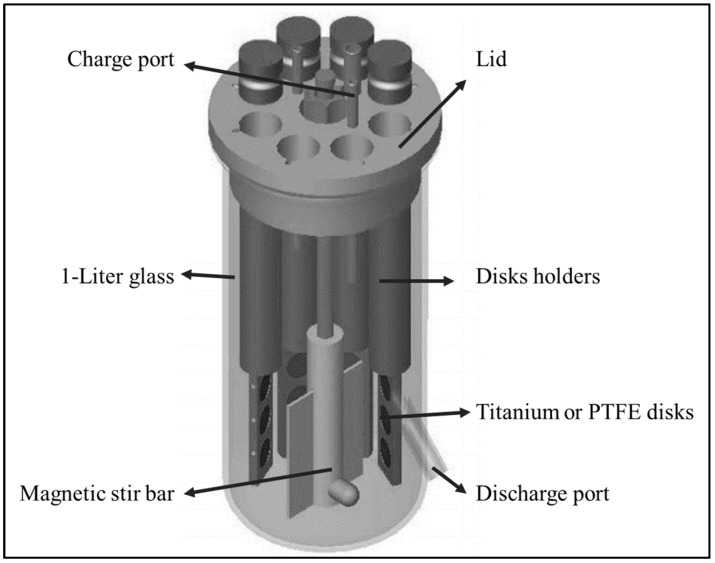
Schematic representation of the CDC Biofilm Reactor. Obtained from BioSurface Technologies Corporation (http://biofilms.biz/).

The CBR has been described elsewhere [18]. Briefly, a CBR (model CBR 90, BioSurface Technologies, Bozeman, MT, USA) consists of eight rods inserted in a ported lid and mounted in a 1-liter glass vessel (Figure 1). Each rod houses three removable disks, which allow a total of 24 sampling opportunities. Disks act as biofilm growth surfaces and have a diameter of 1.27 cm and a height of 0.4 cm. In our case, the CBR was set up with 12 PTFE disks (Model RD128-PTFE) or 12 commercially pure titanium disks (Model RD128-Ti).

### 2.3. Isolate Selection and Characterization

*C. tropicalis* B10, identified by its biochemical properties (YST ID card, Vitek 2, bioMérieux, Marcy-l’Étoile, France), was selected in a previous screening of eight strains isolated from patients with catheter-related *C. tropicalis* bloodstream infection for its highly biofilm-forming capacity at 24 h, determined by crystal violet staining and XTT reduction assay [19,20]. Planktonic MIC of AMB and ANF was determined by the reference CLSI broth microdilution method (M27-A3 document) [21]. ANF and AMB MICs were defined as the lowest drug concentration that caused a reduction of growth ≥50% and 100%, respectively, compared with the control (without antifungal agents). Biofilm MICs (BMIC) were determined by XTT reduction assay as we describe elsewhere [5,22]. The influence of anti-biofilm compounds on the biofilm metabolic activity was assayed by the XTT reduction assay. Cell surface hydrophobicity (CSH) was performed using the water–cyclohexane biphasic assay, previously described, that takes into account the number of cells removed from the aqueous phase [23].

### 2.4. Biofilm Growth Kinetics

Growth kinetics curves were performed in the CBR filled with 400 mL Yeast Nitrogen Base with glucose 100 mM (YNBG). Before the test was performed, the isolate was subcultured at least twice and grown for 24 h on Sabouraud dextrose agar. The inoculum was prepared by suspending one to three colonies in phosphate buffered saline pH 7.3 (PBS) and adjusting cell density to 2 McFarland standard. The CBR was charged with 1 mL of this cell suspension (final concentration in the CBR ranged from 8 × 10^4^ to 2.7 × 10^5^ CFU/mL) and placed on a stir plate with a rotational speed of 125 rpm. CBR was operated for 96 h and was maintained at 30 °C during the experiment. A CBR with 1% of DMSO but without antifungal agent was used as a control for antifungals dissolved in DMSO.

At predetermined time points (24, 48, 72, and 96 h), three disks were removed to ascertain the number of viable cells attached to the surfaces. Disks were rinsed with 14 mL PBS to remove non-adherent cells. Then, each one was placed in 1 mL YNBG, vortexed for 1 min, sonicated at 50 kHz for 1 min, and vortexed again (1 min) to recover the viable cells attached. Samples were then serially diluted and 0.1 mL of each dilution was plated onto brain heart infusion agar and incubated overnight at 37 °C to determine the CFU. The lower limit of accurate and reproducible detectable colony counts in this model was 10 CFU/mL. Results, expressed in CFU/mL, were transformed into Log_10_ CFU/cm^2^ taking into account that the area of each disk is 1.27 cm with two surfaces where biofilms can grow. All time-kill studies were evaluated in triplicate and on at least two different days to ensure reproducibility.

### 2.5. Killing Kinetics of Antifungals plus Anti-Biofilm Compounds

AMB, ANF, RIF, CLA, EDTA, NAC, and FAR alone or combined, were added to the CBR containing 24 h biofilms formed on PTFE or titanium. Before adding the drugs, one rod of PTFE or titanium was extracted from the CBR to determine the number of viable cells attached to its surface at 24 h (time 0). After 24, 48, and 72 h of drug exposure, disks were removed from the holders to establish the number of viable cells attached by using the same protocol as described above. Reductions in colony counts (Δ Log_10_ CFU/cm^2^) at each time point were determined with respect to biofilm control at the same time point. 

### 2.6. Data Analysis

The effect of AMB and ANF combined with anti-biofilm compounds was defined following the established criteria for synergism by time kill curves [24,25]: synergistic when the decrease in CFU/cm^2^ at 72 h was ≥2 Log_10_ compared with that obtained with the antifungal agent alone; improved when the decrease was <2 Log_10_ and ≥1 Log_10_; no interaction when the difference was <1 Log_10_; and antagonistic when there was an increase in CFU/cm^2^ ≥2 Log_10_ compared with that obtained with the antifungal agent alone. Differences among groups were assessed with the analysis of variance (ANOVA) followed by Tukey’s test for multiple comparisons. A *p* value ≤0.05 was considered statistically significant.

## 3. Results

Table 1 shows the susceptibility of *C. tropicalis* to antifungal agents and anti-biofilms compounds. CV staining (OD_550_: 3.42, Cut-off: 0.178) and XTT reduction assay (OD_492_: 1.024) classified this isolate as highly adherent (OD_550_ > 4xCut-off, OD_492_ > 1) following the classification reported by Stepanovic et al. for crystal violet assay and Valentin et al. for XTT reduction assay [19,26]. The CSH was 73.47% (OD_600_ of the control: 0.49; OD_600_ after cyclohexane overlay: 0.13). Biofilm growth kinetics curves showed that *C. tropicalis* was able to colonize both PTFE and titanium surfaces. The number of viable cells in the biofilm control at 0, 24, 48, and 72 h were on PTFE: 4.85 ± 0.2, 4.91 ± 0.6, 4.59 ± 0.19, and 4.57 ± 0.11 Log_10_CFU/cm^2^ and on titanium: 4.24 ± 0.04, 4.23 ± 0.26, 4.22 ± 0.49, and 4.48 ± 0.38 Log_10_CFU/cm^2^, respectively. There was no difference between the number of viable cells present in the biofilms exposed to RIF, EDTA, NAC, CLA, and FAR alone and those present in the biofilm control (data not shown).

### 3.1. Effect of AMB Combined with Anti-Biofilm Compounds on *C. tropicalis* Biofilm Formed on PTFE

**Figure 2:**
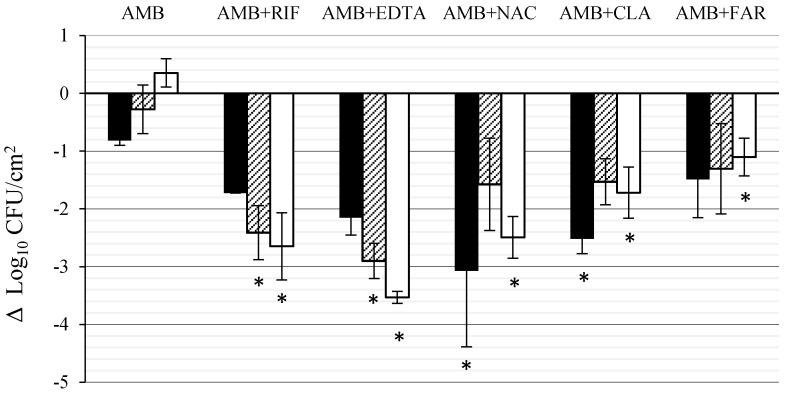
Effect of AMB (1000 mg/L) combined with RIF (1000 mg/L), CLA (1000 mg/L), EDTA (30,000 mg/L), NAC (4000 mg/L) and FAR (300 µM) against *C. tropicalis* biofilm formed on PTFE. Results (Δ Log_10_ CFU/cm^2^) indicate the difference in viable cells at 24 h (black column), 48 h (striped column), and 72 h (white column) of drug exposure with respect to biofilm control at the same time point. Each data point represents the mean and standard deviation for two independent experiments carried out with three replicates. The asterisks indicate that the difference is significant at *p* ≤ 0.05 compared to AMB alone at the same time point. RIF, CLA, EDTA, NAC, and FAR assayed alone, at the same concentrations used when combined, have no anti-biofilm activity (data not shown).

Figure 2 shows the activity of AMB alone and combined with RIF, CLA, EDTA, NAC and FAR against *C. tropicalis* biofilms formed on PTFE disks. AMB alone reduced the viable cells in the biofilm by 0.82 Log_10_ (84.8% killing) at 24 h. From this point, a slow recovery was observed and, at the end of the experiment, the number of viable cells was 0.35 Log_10_ (5.31 ± 0.24 Log_10_CFU/cm^2^), superior to that of time 0. Cell reduction after AMB + EDTA and AMB + RIF exposure increased with time, reaching a maximum Log_10_ reduction of 3.53 Log_10_ (99.9% killing) and 2.65 Log_10_ (99.8% killing), respectively, at the end of the experiment (72 h) this reduction being significant (*p* < 0.05). With AMB + NAC, AMB + CLA and AMB + FAR, the maximum Log_10_ reduction was 3.07, 2.52, and 1.49 Log_10_, respectively (99.9%, 99.7% and 96.7% killing) after 24 h, it being significant (*p* < 0.05) for the first two combinations. It must be noted that while kinetics of the combinations AMB + EDTA and AMB + RIF was linear (*r*^2^ = 0.91 and 0.88, respectively) for the other combinations (AMB + NAC, AMB + CLA, and AMB + FAR) a regrowth in CFU/cm^2^ was observed after 24 h. The interaction of AMB plus RIF or EDTA was synergistic, while CLA or NAC improved the activity of AMB with a tendency to synergism. On the contrary, the interaction of AMB plus FAR was indifferent.

### 3.2. Effect of ANF Combined with Anti-Biofilm Compounds on *C. tropicalis* Biofilm Formed on Titanium

**Figure 3:**
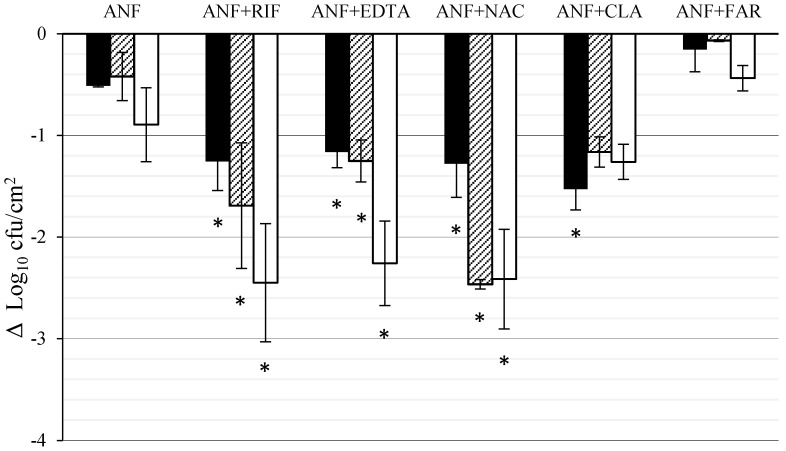
Effect of ANF (8 mg/L) combined with RIF (10 mg/L), CLA (5 mg/L), EDTA (30 mg/L), NAC (40 mg/L) and FAR (3 µM) against *C. tropicalis* biofilm developed on titanium. Results (Δ Log_10_CFU/cm^2^) indicate the difference in viable cells at 24 h (black column), 48 h (striped column), and 72 h (white column) of drug exposure with respect to biofilm control at the same time point. Each data point represents the mean and standard deviation for two independent experiments carried out with three replicates. The asterisks indicate that the difference is significant at *p* ≤ 0.05 compared to AND alone at the same time point. RIF, CLA, EDTA, NAC, and FAR assayed alone, at the same concentrations used when combined, have no anti-biofilm activity (data not shown).

Figure 3 shows the activity of ANF alone and combined with FAR, EDTA, RIF, CLA, and NAC against *C. tropicalis* biofilm developed on titanium disks. ANF alone reached the maximum effect at 72 h (0.9 Log_10_ decrease, 87.3% killing). The combination of ANF + FAR was less active than ANF alone (0.44 Log_10_ decrease, 63.48% killing). The effect ANF + EDTA and ANF + RIF combinations increased linearly with time (*r*^2^ = 0.92 and 0.96, respectively), producing Log_10_ decreases in biofilm viable cells of 2.26 and 2.45 Log_10_ (99.64% and 99.45% killing), respectively, at the end of the experiment. For the combination of ANF + NAC the maximum Log_10_ reduction was obtained after 48 h (2.47 Log_10_, 99.66% killing) and with the combination of ANF + CLA it was reached at 24 h (1.26 Log_10_, 97.03% killing). The reductions in viable cells with respect to those obtained with ANF alone were significant at the three time points (*p* < 0.05), except for the combinations of ANF + CLA and ANF + FAR.

## 4. Discussion

In this work, we have determined the activity of AMB and ANF combined with EDTA, NAC, RIF, CLA and FAR, which have been shown to be effective against bacterial biofilms and, in some cases, against *C. albicans* biofilms [8,9,17,27]. To our knowledge, this is the first study that uses AMB and ANF combined with these compounds against *C. tropicalis* biofilms developed on PTFE and titanium, under continuous stirring conditions. *C. tropicalis* biofilms formed on PTFE were exposed to concentrations usually used in ALT, and those formed on titanium to concentrations reached in serum after the administration of standard doses. The highlights of the present study are that EDTA, NAC, RIF, and CLA enhanced the activity of AMB or ANF against *C. tropicalis* biofilm formed both on PTFE or titanium. Our results strengthen the hypothesis that these anti-biofilm compounds, combined with antifungal agents could be an effective strategy for the treatment of *C. tropicalis* biofilm-related infections.

Some authors have proposed the use of calcium chelating agents, such as EDTA, in combination with antimicrobial agents to eradicate microbial biofilms [28]. This study shows that EDTA, independently of the concentration used, increased the activity of ANF and AMB against *C. tropicalis* biofilm formed both on PTFE or titanium. At the concentration assayed on PTFE (30,000 mg/L) the effect was synergistic, while at the concentration assayed on titanium (30 mg/L) it was improved. Our results allow us to suggest, in accordance with other authors, [29,30] that EDTA, in addition to its anticoagulant properties that can prevent thrombotic occlusions in the lumen of the CVC, also increases the activity of antifungal agents, which could be a novel strategy for the conservative management of CVC-related infections. According to other authors, the increased activity of AMB and ANF in the presence of EDTA could be explained by its ability to sequester the divalent ions essential for the extracellular polymeric matrix structure of biofilms, which enables the penetration of antifungals to the lower layers of biofilm [29]. Also, Ramage et al. [17] have reported that EDTA inhibits the filamentation of *C. albicans* and reduces the expression of HWP1 (adhesin implicated in biofilm formation), producing an unstructured biofilm which consists of a monolayer of adherent cells.

NAC is a small molecule derived from the amino acid cysteine, known for its mucolytic (disruption of disulphide-bonds), antioxidant properties (increases production of glutathione) and as a competitive inhibitor of cysteine [7,31]. Furthermore, its ability to reduce bacterial biofilms when combined with tigecycline or ciprofloxacin has been reported [32]. It also reduces monomicrobial and polymicrobial biofilms formed by *Staphylococcus epidermidis* and *C. albicans* [33]. Our results show that the combinations of NAC with ANF or AMB are more effective than ANF or AMB alone against *C. tropicalis* biofilm, producing more than 2 Log_10_ reduction in viable cells attached to PTFE and titanium compared with the antifungal alone.

RIF, is an antibiotic that inhibits the RNA polymerase, has activity against bacterial biofilms and is used for the treatment of device-related infections [34]. On the other hand, AMB increases cell permeability, which could enable RIF to reach the fungal RNA polymerase and thus explain the synergistic interaction found between AMB and RIF. This mechanism has been suggested by El-Azizi et al. to explain the enhanced activity of this combination against *C. parapsilosis*, *C. krusei*, and *C. glabrata* biofilms [35]. Furthermore, Del Pozo et al., using a different methodology, reported synergistic activity of this combination against biofilms of *C. albicans*, *C. parapsilosis*, and *C. glabrata* developed on microtiter plates, but not against *C. tropicalis* [8]. In our study, we have assayed this combination in a bioreactor under continuous stirring conditions and have found that RIF maintained the biofilm reduction rate along time, without biofilm regrowth, independently of the biomaterial and drug concentration assayed. The discrepancy with Del Pozo et al. could be due to the methodology used. With respect to the combination of ANF + RIF an increase in killing compared to the antifungal alone was observed. The underlying mechanism that might explain this phenomenon is unknown and further studies are needed to clarify this fact.

The role of CLA as an anti-biofilm compound against *Candida* spp. biofilms is not clear. Previous studies have demonstrated that certain enzymatic activities, such as hexosaminidases may be increased in the presence of macrolides and this fact could affect the synthesis of the extracellular matrix [36]. Some authors have found that AMB + CLA shows a synergistic activity against *C. albicans*, *C. parapsilosis* and *C. glabrata* and indifference against *C. tropicalis* [8]. Wu et al. used erythromycin as ALT for the treatment of a CVC-related infection caused by *Staphylococcus aureus* and *C. parapsilosis*, obtaining a clinical improvement after two days [37]. Our in vitro results show that CLA improves the activity of AMB and ANF against *C. tropicalis* biofilm, increasing the killing in more than one Log_10_ both on PTFE and titanium.

FAR is released when there is cell overpopulation, mainly in *C. albicans* biofilms, and acts by inhibiting mycelial development [38,39]. Some authors have reported that FAR reduces the expression of certain surface proteins with hydrophobic domains implicated in the cellular adherence and thus decreasing the CSH of *C. albicans* [40]. Consequently, we considered that FAR, at the concentrations assayed, could improve the AMB and ANF killing, since the CSH of the selected *C. tropicalis* B10 isolate was high (73.47%). Our results show that FAR only improves the activity of AMB, but not that of ANF against *C. tropicalis* biofilms. However, Katragkou et al. reported a synergistic effect of FAR when combined with micafungin and fluconazole, and indifference when combined with AMB against *C. albicans* biofilms by XTT reduction assay [39].

The limitation of this study is that we have assayed only one clinical isolate. However, this isolate has a high biofilm-forming capacity and its BMICs_90_ of AMB and ANF are >8 and >16 mg/L, respectively. Furthermore, we have compared the activity of multiple drug combinations at different concentrations (ALT and serum levels) against biofilms formed on two biomaterials commonly used in medical devices. Moreover, we use an in vitro model (CBR) with continuous stirring that mimics in vivo conditions better than with biofilms formed on 96-well microtiter plates.

## 5. Conclusions

In summary, we show for the first time an enhanced activity of AMB and ANF in the presence of EDTA, NAC, RIF, and CLA and a reduced efficacy of FAR against *C. tropicalis* biofilm formed on PTFE or titanium, using a bioreactor as an in vitro model where the material surfaces are under continuous stirring conditions. Although further studies are required to confirm these observations, our findings suggest that anti-biofilm compounds could be a new effective strategy for the treatment of *C. tropicalis* biofilm-related infections.

## Figures and Tables

**Table 1:** Susceptibility of *C. tropicalis* B10 to drug assays.

**Planktonic MIC**	**mg/L**
Amphotericin B	0.25
Anidulafungin	0.03
**Biofilm MIC 50/90**	
Amphotericin B	0.25/8
Anidulafungin	>16/>16
Ethylenediaminetetraacetic acid	>1000/>1000
Rifampicin	>20/>20
*N*-acetylcysteine	>80/>80
Clarithromycin	>128/>128
Farnesol (µM)	>200/>200

Biofilm MIC 50/90: Lowest concentration producing 50%/90% metabolic inhibition with respect to control.
